# Long-term sequelae of severe sepsis: cognitive impairment and structural brain alterations – an MRI study (LossCog MRI)

**DOI:** 10.1186/1471-2377-14-145

**Published:** 2014-07-15

**Authors:** Theresa Götz, Albrecht Günther, Otto W Witte, Frank M Brunkhorst, Gundula Seidel, Farsin Hamzei

**Affiliations:** 1 Biomagnetic Center, Hans Berger Department of Neurology, Jena University Hospital, Jena, Germany; 2 Hans Berger Department of Neurology, Jena University Hospital, Jena, Germany; 3 Integrated Research and Treatment Center, Center for Sepsis Control and Care (CSCC), Jena University Hospital, Jena, Germany; 4 Paul-Martini-Clinical Sepsis Research Unit, Department of Anaesthesiology and Intensive Care Medicine, Jena University Hospital, Jena, Germany; 5 Moritz Klinik GmbH & Co. KG, Department of Neurology, Bad Klosterlausnitz, Germany

**Keywords:** Magnetoencephalography (MEG), Magnet resonance imaging (MRI), fMRI, Neuropsychological tests, Severe sepsis and septic shock

## Abstract

**Background:**

The number of patients with cognitive impairment after sepsis or septic shock is high. However, the underlying neurophysiological basis of sepsis induced cognitive impairment is not fully understood.

**Methods/Design:**

This is a prospective, controlled observational study. We are in the process of recruiting 25 survivors of severe sepsis or septic shock who will be investigated with functional MRI (fMRI), T1-weighted MRI und Diffusion Tensor Imaging (DTI) as well as Magnetoencephalography (MEG). Furthermore, patients will undergo neuropsychological evaluation using the DemTect and the clock drawing tests. In addition, verbal and declarative memory is assessed by the Verbal Learning and Memory Test. The primary aim is to determine the volumetry of the amygdala and the hippocampus. The secondary aim is to analyze the relationship between cognitive tests and MEG, and the (f)MRI results. Moreover, a between-group comparison will be evaluated to an age-matched group of healthy controls.

**Discussion:**

In a previous MEG study, we observed a significant slowing of the prominent background activity in sepsis survivors and hepatic encephalopathy patients in particular shortly after discharge from the ICU. Intriguingly, the rhythmic brain activity after visual flickering stimulation was altered in sepsis survivors in comparison to age-matched healthy volunteers. We propose that this desynchronization is based on affected underlying neuronal responses between various interconnected brain regions. The current project will analyze whether the modifications are related to a damage of the fibers connecting different brain regions or to a disturbance of the functional interaction between different brain regions or even due to an atrophy of certain brain regions.

**Trial registration:**

“Langzeitfolgen nach schwerer Sepsis: Kognitive Beeinträchtigungen und strukturelle Veränderungen am Gehirn, eine MRT Studie”; German Clinical Trials Register (DRKS00005484).

## Background

Since the study conducted by Iwashyna [1], long term sequelae following sepsis have gained increasing attention. Results from the study demonstrate that the long term rate and severity of functional and cognitive decline significantly increase after sepsis [1].

In our recent MEG study, we found a desynchronization of brain function after sepsis in comparison to age-matched healthy volunteers. This desynchronization was associated with impaired cognitive function (Götz et al., under preparation). We suggest that this desynchronization is based on an altered functional interaction between certain brain regions. We believe that either the connections are impaired or an atrophy of these brain regions has occurred. Structural changes in patients surviving intensive care were firstly reported by Semmler [2]. In comparison to healthy controls, he described a significant volume reduction of the hippocampus in sepsis survivors. This was accompanied by a reduced verbal and learning ability during a period of 6–24 months post sepsis. To analyze these circumstances in the current multimodal imaging study of LOSS-Cog MRI, cognitively impaired sepsis survivors will be investigated using magnetoencephalography (MEG), functional MRI (fMRI), T1-weighted MRI and Diffusion Tensor Imaging (DTI). We are particularly interested in the underlying neurophysiological mechanisms of sepsis- or septic shock induced cognitive impairment.

### Objective

In a group of 25 sepsis survivors with long-lasting cognitive deficits after sepsis, we aim to investigate functional status using MEG, fMRI, and structural brain organization via analysis of connecting fibers and volumetry of the connecting regions. We assume that cognitive deficits could be based on altered brain organization as a “disconnection syndrome” with deterioration of nodes and/or damage of distributed tracts connecting these nodes. Although cognitive impairments are now generally accepted as long-lasting sequelae after severe sepsis [1], knowledge regarding disconnection and relation to functional status such as rhythmic brain activity in former sepsis patients is still lacking.

## Methods/Design

### Study design

The study is a prospective, controlled observational study at the Center for Sepsis Control and Care (CSCC) and the Biomagnetic Center of the Hans Berger Department of Neurology, University Hospital Jena.

### Study population

Survivors of severe sepsis or septic shock who requested gratis advice from the German Sepsis Aid’s National Helpline between 2011 and 2013 are included. Neurocognitive impairment is currently assessed by the Informant Questionnaire on Cognitive Decline in the Elderly (IQCODE) and persons with a value above 3.29 are included into the study. The age range of all participants lies between 18–65 years (valid age during sepsis).

We exclude participants with known cerebral impairments due to ischemia and/or other CNS disorders. Since MEG and MRI measurements are part of the study, participants with cardiac pacemakers and other electromagnetic devices are excluded as well as blind participants. Moreover, participants who are currently on treatment with neuroleptics, anesthetics and antidepressants cannot take part in the study.

### Outcome measures

Primary outcome measures will be based on the results of the neurophysiological assessment of patients in terms of MEG and MRI.

#### Behavioral outcomes

We collect behavioral data from cognitive testing in terms of the DemTect [3] and clock drawing test scores.

Moreover, verbal and declarative memory (learning and long-term encoding and retrieval) are assessed by the Verbal Learning and Memory Test (VLMT, Helmstaedter, Lendt und Lux, Beltz Test GmbH, Göttingen 2001).

A major part of the study is TAP (Test of Attentional Performance “Testbatterie für Aufmerksamkeitsprüfung”). On the one hand, we assess tonic alertness in which maintenance of a high level of reaction and capacity over a long period of time is required. During the Go/No-Go paradigm, the suppression of unwanted reactions is tested, whereas the split attention paradigm analyzes the simultaneous execution of a visual and an auditory task.

#### Neurophysiological (MEG)

During MEG measurement, we assess the prominent resting frequency in Hz with open or closed eyes without any stimulation. In another experiment, we generate artificial neuronal oscillations with the help of visual flickering stimulation. With the flicker coupling score [4] derived from our MEG data, we can determine the adaptation of the brain towards the flickering stimulus.

#### Neurophysiological (3 T MRI)

We acquire a T1-weighted MRI. Using the method of voxel-based morphometry (VBM), brain volumes can be analyzed (thalamus, hippocampus and amygdale). It is also possible to determine the dimension of brain atrophy. The method of diffusion tensor imaging (DTI) will shed some light on possible damaged fibers. The extent of damage can be determined via fractional anisotropy (FA), a method measuring the diffusion coefficient along a fiber. Moreover, we will analyze the connectivity between different regions of interest (ROI).

#### Primary endpoints

– measure of brain atrophy determined as ventricle brain ratio

volumetry of the amygdala and the hippocampus.

– Flicker coupling index as determined by the MEG

#### Secondary endpoints

– relationship between cognitive tests and MEG parameters

– relationship between cognitive tests and MRI related data: fMRI with analysis of the functional connectivity between regions of interest

– relationship between FA of DTI and VBM with cognitive tests

– Difference between patients and control group regarding MEG und MRI results

### Methods against bias

MEG and (f)MRI data are analyzed independently and blinded from cognitive testing, which will be conducted and evaluated by trained neuropsychologists.

### Sample size

In a previous feasibility study, we collected data from > 30 healthy controls, 25 patients with liver cirrhosis, and 25 severe sepsis patients. Despite the small patient groups, differences between the two groups were more than obvious in terms of flicker frequency, and resting and evoked activity. The proposed number of participants lies well within the dimension of common MEG/MRI studies in this field.

With the help of G*Power 3.1.5 (Franz Faul, University of Kiel), we calculated the sample size needed for a two-tailed correlation between cognitive score and amygdala size. Using an α-error of 5% and assuming a correlation of 0.6, we would need 26 patients to find a correlation with a probability of 95%.

### Feasibility of recruitment

We intended to use the study cohort from the German Sepsis Aid’s (GSA) National Helpline (http://www.sepsis-hilfe.org/). The GSA (Office: Paul-Martini-Research Group, c/o Jena University Hospital) is a non-profit self-help group for sepsis survivors with outstanding experience in long-term quality-of-life evaluations and personal counseling of survivors of critical illness via an established national hotline available 12 hours a day throughout Germany since 2005. More than 2.000 contact addresses are available in the database of the organization. As we anticipated, a majority of former sepsis patients who regularly communicate with the GSA office are highly interested in participating in our study. Sepsis survivors were contacted via email and by phone through the GSA office in order to ensure timely recruitment. Where required, participants’ travel expenses will be reimbursed.

### Ethical considerations

MEG measurements are completely non-invasive and the MRI investigation will be free of risks under consideration of standard safety instructions. The study is conducted according to the local legislation and was approved by the ethics committee of the University Hospital Jena (reference number: 3853-08/13). All patients will be informed that they can withdraw their consent of participation and request deletion of their data at any time during the course of the study.

The study was registered at the German Clinical Trials Register (DRKS00005484).

### Recruitment

The recruitment and observation of sepsis survivors started in September 2013. All study related activities follow the study protocol. It is planned to finish recruitment in May 2015.

## Discussion

In a group of 25 sepsis survivors with long-lasting cognitive deficits after sepsis, we investigate patients’ functional status using MEG and fMRI. We also gain anatomical information from the MRI.

In MEG, functional impairments were already reported in a previous feasibility study by our group (“NeuroSOS-Sync”). In the named study, we included a cohort of 25 liver cirrhosis patients because cognitive impairments (“hepatic encephalopathy”) are well documented in this patient group. We observed a significant slowing of the prominent background activity in liver cirrhosis patients. The same result was found in sepsis survivors, who showed the lowest resting frequency shortly after ICU discharge. Intriguingly, the resting frequency gradually increased over several months following ICU discharge in our sepsis survivors. The same reduction of the alpha frequency, which even seemed to be paralleled by a reduction of regional cerebral blood flow, was also reported in septic rats [5].

Moreover, we also showed impairments in rhythmic brain activity after visual flickering stimulation in patients with liver cirrhosis and sepsis survivors. Impairments in the visual system have already been described in liver cirrhosis patients at the behavioral level [6]. When exposed to a flickering stimulus, (occipital) brain waves are entrained at the stimulation frequency [7]. Compared to healthy controls, this ability was severely impaired in patients with liver cirrhosis. They showed a broader frequency band with no dynamical adaptation to the flickering stimulation (entrainment at the signal when flickering, no signal during pause) and a weaker coupling to the flickering stimulation. We obtained an individual score to describe the aberrance of frequency coupling in each study participant, which revealed a significantly decreased score in patients with liver cirrhosis [4].

We suggest that the temporal coordination of neuronal responses is affected and causes a desynchronization between various interconnected brain regions. This could also be linked to a reduction in cerebral blood flow. In Alzheimer’s disease, for example, a similar visual cortical dysfunction in terms of a reduction in cerebral blood flow was detected via PET [8]. In the latter study, cerebral blood flow in response to flickering stimuli was measured at 1, 2, 4, 7 and 14 Hz. At 7 and 14 Hz, regional cerebral blood flow in the striate cortex was found to be reduced in patients in comparison to healthy controls. More interestingly, in sepsis survivors, our score positively correlated to the performance in the DemTect and to the time after ICU discharge. These results imply that cognitive impairments in patients after sepsis are related to a disturbed functional interaction between regions. Alternatively, such a disturbance might be based on structural damage following sepsis.

## Abbreviations

ARDS: Acute respiratory distress syndrome; CNS: Central nervous system; DemTect: Detection of dementia; DRKS: German clinical trials register (“Deutsches Register klinischer Studien”); DTI: Diffusion tenor imaging; FA: Fractional anisotropy; GSA: German sepsis aid; ICU: Intensive care unit; IQCode: Informant questionnaire on cognitive decline in the elderly; MEG: Magnetoencephalography; (f)MRI: (functional) magnetic resonance imaging; PET: Positron emission tomography; ROI: Region of interest; VBM: Voxel based morphometry.

## Competing interests

The authors declare that they have no competing interests.

## Authors’ contributions

TG, AG, FMB, FH and OW developed the concept and design, AG is the trial physician while TG and GS acquire and analyze the data together with FH. All authors read and approved the final manuscript.

## Pre-publication history

The pre-publication history for this paper can be accessed here:

http://www.biomedcentral.com/1471-2377/14/145/prepub
